# Employment and Activity Limitations Among Adults with Chronic Obstructive Pulmonary Disease — United States, 2013

**Published:** 2015-03-27

**Authors:** Anne G. Wheaton, Timothy J. Cunningham, Earl S. Ford, Janet B. Croft

**Affiliations:** 1 Division of Population Health, National Center for Chronic Disease Prevention and Health Promotion, CDC

Chronic obstructive pulmonary disease (COPD) is a group of progressive respiratory conditions, including emphysema and chronic bronchitis, characterized by airflow obstruction and symptoms such as shortness of breath, chronic cough, and sputum production. COPD is an important contributor to mortality and disability in the United States (1,2). *Healthy People 2020* has several COPD-related objectives,* including to reduce activity limitations among adults with COPD. To assess the state-level prevalence of COPD and the association of COPD with various activity limitations among U.S. adults, CDC analyzed data from the 2013 Behavioral Risk Factor Surveillance System (BRFSS). Among U.S. adults in all 50 states, the District of Columbia (DC), and two U.S. territories, 6.4% (an estimated 15.7 million adults) had been told by a physician or other health professional that they have COPD. Adults who reported having COPD were more likely to report being unable to work (24.3% versus 5.3%), having an activity limitation caused by health problems (49.6% versus 16.9%), having difficulty walking or climbing stairs (38.4% versus 11.3%), or using special equipment to manage health problems (22.1% versus 6.7%), compared with adults without COPD. Smokers who have been diagnosed with COPD are encouraged to quit smoking, which can slow the progression of the disease (3) and reduce mobility impairment (4). In addition, COPD patients should consider participation in a pulmonary rehabilitation program that combines patient education and exercise training to address barriers to physical activity, such as respiratory symptoms and muscle wasting (5).

Each year, the BRFSS survey is administered by state health departments in collaboration with CDC. BRFSS is a random-digit–dialed telephone survey (landline and cell phone) of noninstitutionalized civilian adults aged ≥18 years that includes various questions about respondents’ health and risk behaviors. Response rates for BRFSS are calculated using standards set by the American Association of Public Opinion Research Response Rate Formula #4.† The response rate is the number of respondents who completed the survey as a proportion of all eligible and likely eligible persons. The median survey response rate for all states, territories, and DC in 2013 was 46.4%, and ranged from 29.0% to 60.3%. Additional information is presented in the BRFSS 2013 Summary Data Quality Report.§

Self-reported, physician-diagnosed COPD was defined as a positive response to the question, “Have you ever been told by a doctor or health professional that you have COPD, emphysema, or chronic bronchitis?” Several questions addressed activity limitations: “Are you limited in any way in any activities because of physical, mental, or emotional problems?”; “Do you have serious difficulty walking or climbing stairs?”; and “Do you now have any health problem that requires you to use special equipment, such as a cane, a wheelchair, a special bed, or a special telephone?” Being unable to work was defined for respondents who reported they were unable to work in response to the question, “Are you currently…? Employed for wages, self-employed, out of work for 1 year or more, out of work for less than 1 year, a homemaker, a student, retired, or unable to work.” Current smokers reported having smoked at least 100 cigarettes in their life and currently smoking cigarettes some days or every day. Former smokers reported having smoked at least 100 cigarettes in their life but were not current smokers. Respondents were categorized as engaging in physical activity if they answered “yes” to the question, “During the past month, other than your regular job, did you participate in any physical activities or exercises such as running, calisthenics, golf, gardening, or walking for exercise?”

The age-adjusted prevalence of self-reported, physician-diagnosed COPD (with 95% confidence intervals) was calculated by state, selected demographic characteristics, smoking status, physical activity status, and activity limitation characteristics. Additionally, the age-adjusted prevalence of activity limitation measures was calculated by COPD status, current smoking status, and physical activity status. T-tests were used to compare prevalence between subgroups (significance at p<0.05). All indicated differences between subgroups are statistically significant. Data are weighted to state population estimates, and statistical software that took into account the complex sampling design was used.

Overall, 6.4% of U.S. adults (an estimated 15.7 million) were told by a physician or other health care provider that they have COPD (age-adjusted prevalence = 6.0%) (Table). Prevalence of COPD ranged from 2.6% among those aged 18–34 years to 12.3% among those aged ≥75 years. In age-adjusted comparisons by race/ethnicity, Asians were the least likely to report COPD (2.0%), whereas adults who identified themselves as multiracial or American Indian/Alaska Native reported the highest prevalence (10.7% and 10.2%, respectively). Women were more likely to report COPD than men (6.6% compared with 5.4%). COPD prevalence was lower among employed adults (3.6%) compared with other employment categories. COPD prevalence was lower with greater educational level. COPD also varied by marital status, with divorced, widowed, or separated respondents being more likely to report COPD (9.1%) than married respondents (4.7%). COPD was more common among current smokers (14.3%) than former smokers (7.0%) or never smokers (2.8%) and among respondents who reported not exercising in the past month compared with those who had exercised (8.8% versus 4.9%). COPD was also more common among those who reported each of the activity limitation measures: health-related activity limitation (15.1% versus 3.6%), difficulty walking or climbing stairs (18.2% versus 3.9%), use of special equipment (18.7% versus 4.9%), and being unable to work (20.4% versus 4.8%). State-specific prevalence of COPD ranged from 3.6% in Puerto Rico and 4.0% in Minnesota and South Dakota to >9% in West Virginia (9.4%), Alabama (9.6%), and Kentucky (10.3%). COPD prevalence was highest for states along the Ohio and lower Mississippi rivers (Figure 1).

More than one third (38.0%) of adults with COPD were current smokers. Activity limitations were common among adults with COPD. Adults who reported having COPD were more likely to report being unable to work (24.3% versus 5.3% for adults without COPD), having activity limitation because of health problems (49.6% versus 16.9%), having difficulty walking or climbing stairs (38.4% versus 11.3%), and use of special equipment for health problems (22.1% versus 6.7%) compared with adults without COPD. Among adults with COPD, nonsmokers who also reported being physically active were least likely to report all of the activity limitation measures (Figure 2), whereas those not physically active, regardless of smoking status, were most likely to report the activity limitations.

## Discussion

COPD is an important contributor to both mortality and disability in the United States (1,2). COPD is the primary contributor (>95%) to deaths from chronic lower respiratory diseases, the third leading cause of death in the United States (1). Among diseases and injuries, COPD also is the sixth largest contributor to number of years lived with disability in the United States (2). COPD is costly, with COPD-related medical costs estimated at $32 billion in the United States in 2010 and an additional $4 billion in absenteeism costs (6). Persons with COPD are less likely to be employed and more likely to be limited in the type of work they can do compared with persons without COPD (7).

In this study, adults with COPD were more likely to report activity limitations and being unable to work compared with adults without COPD. COPD has been found to be associated with a lower likelihood of employment, comparable with that for stroke and greater than that associated with heart disease or hypertension (8). After accounting for age, U.S. adults with COPD are also more likely to collect Social Security Disability Insurance and Supplemental Security Income than those without the condition (8). Together, these results underscore the substantial economic burden of COPD, which only adds to the impaired quality of life experienced by persons with COPD. Because there is currently no cure for COPD, public health efforts should focus on prevention, such as antismoking efforts, and treatment to slow the progression of the disease, manage comorbidities, and lessen symptoms (9).

Smoking, the leading cause of COPD in the United States, is also associated with worse symptoms among persons with COPD (10), and smoking cessation has been shown to slow the progression of COPD (3). Among adults with COPD in these analyses, more than one third were current smokers. Current smoking was associated with a greater likelihood of three of the four activity limitations measured among those who reported being physically active. This result reinforces the importance of smoking cessation by COPD patients. Health care providers play a critical role in motivating and assisting their patients, including those with COPD, with smoking cessation. Information for health care providers on helping patients quit smoking is available online.¶ Quitting resources for patients also are available.**

Not being physically active was associated with a greater likelihood of all the activity limitation measures among persons with COPD. This association might indicate that COPD affects patients’ ability to be physically active, but not being physically active might also reinforce activity limitations. Although respiratory symptoms such as shortness of breath can cause activity limitations, COPD is also associated with muscle weakness, which can also contribute to limited mobility (5). Although physical activity might be challenging for persons with COPD, exercise training is an essential part of pulmonary rehabilitation (5). Pulmonary rehabilitation is a personalized program that includes both education and exercise components to improve management of breathing problems, increase stamina, and decrease shortness of breath. These programs should incorporate both strength and endurance (or aerobic) training. Patients can learn more about pulmonary rehabilitation online.†† Physicians should refer to the latest clinical practice guidelines (5).

The findings in this report are subject to at least three limitations. First, COPD diagnosis relied on self-report and not on evaluation by breathing tests or review of medical records. Second, this was a cross-sectional study; therefore, it is not possible to determine whether the COPD or activity limitations came first. Finally, state response rates ranged from 29.0% to 60.3%; therefore, nonresponse bias might have affected the results.

COPD is strongly associated with activity limitations and an inability to work. Current smoking and lack of physical activity were both associated with greater percentages reporting activity limitation and inability to work among those with COPD. COPD patients who smoke should be encouraged to quit and provided with the support they need to achieve this objective, whereas all COPD patients might benefit from pulmonary rehabilitation and a personalized exercise regimen. Outside the clinical setting, the development of state and community environmental and policy efforts to address smoking and physical inactivity could improve outcomes for persons with COPD as well as for the general population. CDC’s *Best Practices for Comprehensive Tobacco Control Programs—2014* is an evidence-based guide to help states develop and implement tobacco control programs.§§ CDC also has compiled a guide to community-based strategies to increase physical activity: *The CDC Guide to Strategies to Increase Physical Activity in the Community.*¶¶

**What is already known on this topic?**

Chronic obstructive pulmonary disease (COPD) is a group of progressive respiratory conditions, including emphysema and chronic bronchitis, characterized by airflow obstruction and symptoms such as shortness of breath, chronic cough, and sputum production. COPD is an important contributor to mortality and disability in the United States.

**What is added by this report?**

Adults who reported having COPD were more likely to report being unable to work (24.3% versus 5.3%), activity limitation resulting from a health problem (49.6% versus 16.9%), difficulty walking or climbing stairs (38.4% versus 11.3%), and use of special equipment for health problems (22.1% versus 6.7%) compared with adults without COPD. Among adults with COPD, nonsmokers who also reported being physically active were least likely to report all of the activity limitation measures, whereas those who were inactive, regardless of smoking status, were most likely to report the activity limitations.

**What are the implications for public health practice?**

COPD patients who smoke should be encouraged to quit and provided with the support they need to achieve this objective, whereas all COPD patients might benefit from pulmonary rehabilitation and a personalized exercise regimen.

## Figures and Tables

**FIGURE 1:**
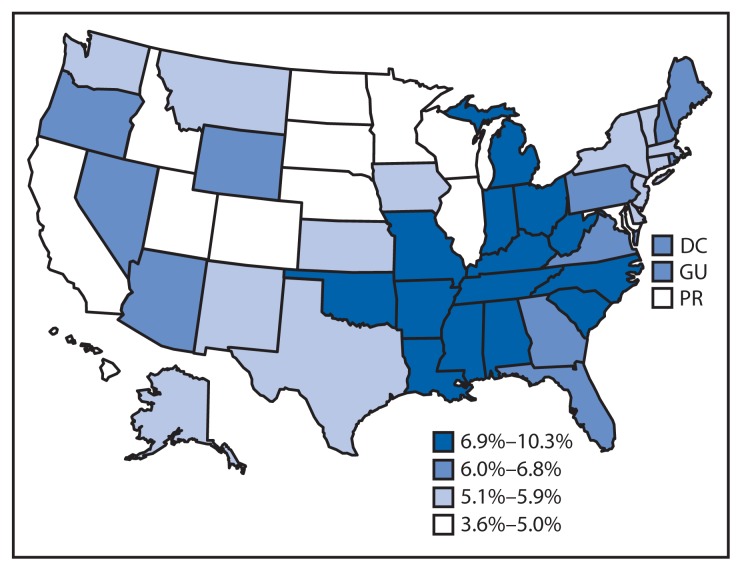
Age-adjusted prevalence* of chronic obstructive pulmonary disease (COPD)^†^ among adults aged ≥18 years —Behavioral Risk Factor Surveillance System, United States, 2013 * Age-adjusted to the 2000 U.S. standard population aged ≥18 years. ^†^ Based on a positive response to the question, “Have you ever been told by a doctor or health professional that you have COPD, emphysema, or chronic bronchitis?”

**FIGURE 2:**
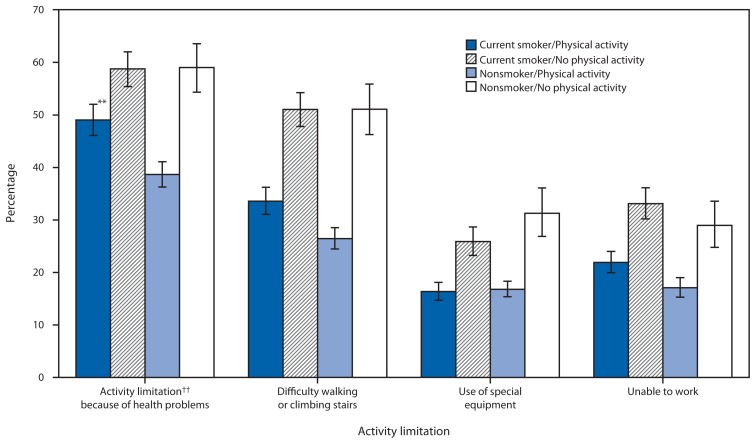
Age-adjusted percentage* of adults with chronic obstructive pulmonary disease (COPD)^†^ aged ≥18 years with activity limitations, by smoking^§^ and physical activity^¶^ status — Behavioral Risk Factor Surveillance System, United States, 2013 ^*^ Age-adjusted to the 2000 U.S. standard population aged ≥18 years. ^†^ Based on a positive response to the question, “Have you ever been told by a doctor or health professional that you have COPD, emphysema, or chronic bronchitis?” ^§^ Current smokers reported smoking ≥100 cigarettes in their life and currently smoking cigarettes some days or every day. Nonsmokers include former smokers and never smokers. ^¶^ Respondents were categorized as engaging in physical activity if they answered “yes” to the question, “During the past month, other than your regular job, did you participate in any physical activities or exercises such as running, calisthenics, golf, gardening, or walking for exercise?” ^**^ 95% confidence interval. ^††^ Respondents were categorized as having activity limitations if they answered “yes” to the question, “Are you limited in any way in any activities because of physical, mental, or emotional problems?”

**TABLE:** Age-adjusted* percentage of adults aged ≥18 years reporting having ever been told by a physician that they had chronic obstructive pulmonary disease (COPD)†, by selected characteristics — Behavioral Risk Factor Surveillance System, United States, 2013

Characteristic	No.	%§	(95% CI)	Estimated no.¶ with COPD
**Total respondents (crude)**	**486,921**	**6.4**	**(6.3–6.5)**	**15,667,000**
**Total (age-adjusted)**	**486,921**	**6.0**	**(5.9–6.1)**	
**Age group (yrs) (unadjusted)**				
18–34	77,294	2.6	(2.4–2.8)	1,931,000
35–44	59,556	3.6	(3.3–3.9)	1,447,000
45–54	83,324	6.7	(6.4–7.1)	2,976,000
55–64	106,090	9.7	(9.3–10.0)	3,823,000
65–74	89,992	11.8	(11.4–12.2)	3,061,000
≥75	70,665	12.3	(11.8–12.8)	2,429,000
**Race/Ethnicity**				
White, non-Hispanic	373,527	6.3	(6.2–6.5)	11,237,000
Black, non-Hispanic	38,686	6.5	(6.1–6.9)	1,844,000
American Indian/Alaska Native, non-Hispanic	7,626	10.2	(8.8–11.7)	267,000
Asian, non-Hispanic	9,381	2.0	(1.4–2.9)	181,000
Native Hawaiian/Pacific Islander, non-Hispanic	1,531	6.2	(3.1–12.0)	30,000
Other race, non-Hispanic	2,627	4.8	(3.8–5.9)	44,000
Multiracial, non-Hispanic	9,059	10.7	(9.2–12.4)	321,000
Hispanic	36,826	4.1	(3.7–4.5)	1,414,000
**Sex**				
Men	199,660	5.4	(5.2–5.6)	6,679,000
Women	287,261	6.6	(6.4–6.8)	8,988,000
**Employment status**				
Employed	239,796	3.6	(3.5–3.8)	4,352,000
Unemployed	26,081	8.2	(7.6–8.9)	1,351,000
Homemaker	31,367	4.5	(4.1–5.0)	782,000
Student	12,602	7.5	(5.1–10.8)	309,000
Retired	136,906	8.7	(6.6–11.4)	4,714,000
Unable to work	37,170	20.4	(19.3–21.4)	4,067,000
**Education level**				
Less than high school diploma or GED	41,949	9.8	(9.3–10.3)	3,898,000
High school diploma or GED	141,867	6.8	(6.6–7.0)	5,145,000
At least some college	301,281	4.6	(4.5–4.8)	6,556,000
**Marital status**				
Married	251,036	4.7	(4.5–4.9)	6,924,000
Divorced/Widowed/Separated	145,601	9.1	(8.7–9.5)	5,822,000
Member of unmarried couple	74,536	6.9	(6.4–7.3)	2,339,000
Never married	13,127	7.0	(6.1–8.1)	511,000
**Smoking status** **				
Current smoker	76,266	14.3	(13.8–14.8)	5,754,000
Former smoker	137,125	7.0	(6.7–7.3)	5,653,000
Never smoker	258,811	2.8	(2.7–2.9)	3,752,000
**Physical activity** ††				
Yes	329,512	4.9	(4.8–5.0)	8,327,000
No	124,313	8.8	(8.5–9.1)	6,159,000
**Activity limitation due to health problems** §§				
Yes	115,869	15.1	(14.6–15.6)	8,430,000
No	361,933	3.6	(3.5–3.7)	6,867,000
**Difficulty walking or climbing stairs**				
Yes	85,876	18.2	(17.4–19.0)	7,237,000
No	389,975	3.9	(3.8–4.1)	8,019,000
**Use of special equipment**				
Yes	55,401	18.7	(17.6–19.8)	4,415,000
No	421,944	4.9	(4.8–5.0)	10,874,000
**Unable to work**				
Yes	37,170	20.4	(19.3–21.4)	4,067,000
No	446,752	4.8	(4.7–4.9)	11,509,000
**State/Area**				
Kentucky	10,933	10.3	(9.5–11.2)	367,000
Alabama	6,450	9.6	(8.6–10.8)	382,000
West Virginia	5,853	9.4	(8.5–10.3)	155,000
Tennessee	5,750	8.7	(7.8–9.7)	473,000
Mississippi	7,401	8.4	(7.5–9.3)	195,000
Arkansas	5,208	8.2	(7.3–9.3)	200,000
Michigan	12,646	7.9	(7.2–8.5)	661,000
Ohio	11,851	7.6	(7.0–8.3)	737,000
Indiana	10,237	7.5	(6.9–8.1)	394,000
South Carolina	10,601	7.3	(6.7–8.0)	292,000
Oklahoma	8,202	7.3	(6.7–8.0)	227,000
Louisiana	5,207	7.2	(6.3–8.2)	261,000
Missouri	7,056	7.1	(6.3–8.0)	351,000
North Carolina	8,768	6.9	(6.2–7.6)	556,000
Rhode Island	6,455	6.8	(6.0–7.8)	61,000
Arizona	4,205	6.8	(5.3–8.6)	350,000
Wyoming	6,370	6.6	(5.9–7.4)	32,000
Florida	33,776	6.4	(5.9–7.0)	1,139,000
Pennsylvania	11,303	6.4	(5.8–7.0)	712,000
New Hampshire	6,383	6.4	(5.6–7.2)	74,000
Virginia	8,374	6.3	(5.7–7.0)	422,000
Nevada	5,047	6.3	(5.2–7.6)	142,000
Georgia	8,051	6.2	(5.6–6.9)	485,000
Maine	8,031	6.1	(5.5–6.8)	75,000
District of Columbia	4,841	6.0	(5.1–7.2)	31,000
Oregon	5,908	6.0	(5.3–6.8)	199,000
Guam	1,875	6.0	(4.6–7.9)	6,000
Kansas	23,135	5.8	(5.5–6.2)	135,000
Montana	9,638	5.8	(5.2–6.5)	51,000
Iowa	8,094	5.8	(5.2–6.5)	149,000
Alaska	4,533	5.6	(4.7–6.7)	30,000
New Mexico	9,224	5.5	(4.9–6.2)	93,000
Delaware	5,150	5.5	(4.8–6.3)	43,000
New Jersey	13,179	5.4	(4.9–6.0)	400,000
Texas	10,783	5.3	(4.7–5.9)	1,040,000
Connecticut	7,609	5.3	(4.7–6.0)	163,000
Washington	11,065	5.3	(4.8–5.8)	301,000
New York	8,805	5.2	(4.7–5.9)	856,000
Massachusetts	14,914	5.1	(4.7–5.7)	296,000
Vermont	6,322	5.1	(4.5–5.8)	28,000
Maryland	12,830	5.0	(4.5–5.6)	244,000
Wisconsin	6,521	5.0	(4.2–5.8)	245,000
Nebraska	17,017	4.9	(4.4–5.3)	74,000
Illinois	5,586	4.8	(4.1–5.5)	491,000
California	11,507	4.5	(4.1–5.0)	1,352,000
North Dakota	7,725	4.5	(3.9–5.1)	27,000
Colorado	13,487	4.4	(4.0–4.9)	182,000
Hawaii	7,788	4.4	(3.7–5.1)	51,000
Utah	12,648	4.2	(3.8–4.6)	80,000
Idaho	5,573	4.2	(3.6–4.9)	52,000
South Dakota	6,859	4.0	(3.4–4.7)	28,000
Minnesota	14,180	4.0	(3.4–4.6)	175,000
Puerto Rico	5,967	3.6	(3.1–4.2)	104,000
**Median (range)**		**6.0**	**(3.6–10.3)**	

**Abbreviations:** CI = confidence interval; GED = General Education Development certificate.

* Age-adjusted to the 2000 U.S. standard population aged ≥18 years.

† Includes emphysema and chronic bronchitis.

§ Weighted percentage.

¶ Numbers might not add to total because of rounding.

** Current smokers smoked ≥100 cigarettes in their life and currently smoking cigarettes some days or every day. Former smokers smoked ≥100 cigarettes in their life but were not current smokers. Never smokers did not smoke ≥100 cigarettes in their life.

†† Respondents were categorized as engaging in physical activity if they answered “yes” to the question, “During the past month, other than your regular job, did you participate in any physical activities or exercises such as running, calisthenics, golf, gardening, or walking for exercise?”

§§ Respondents were categorized as having activity limitations if they answered “yes” to the question, “Are you limited in any way in any activities because of physical, mental, or emotional problems?”

## Footnotes

* Additional information available at http://www.healthypeople.gov/2020/topics-objectives/topic/respiratory-diseases/objectives?topicId=36.

† Available at http://www.aapor.org/AAPORKentico/AAPOR_Main/media/MainSiteFiles/StandardDefinitions2011_1.pdf.

§ Available at http://www.cdc.gov/brfss/annual_data/2013/pdf/2013_DQR.pdf.

¶ Available at http://www.ahrq.gov/professionals/clinicians-providers/guidelinesrecommendations/tobacco/index.html and at http://www.cdc.gov/tobacco/campaign/tips/partners/health/hcp.

** Available at http://www.cdc.gov/tobacco/campaign/tips/quit-smoking/quitting-resources.html.

†† Available at http://www.thoracic.org/patients/patient-resources/resources/pulmonary-rehab.pdf.

§§ Available at http://www.cdc.gov/tobacco/stateandcommunity/best_practices/index.htm.

¶¶ Available at http://www.cdc.gov/obesity/downloads/PA_2011_WEB.pdf.
